# Associations between mass media exposure and birth preparedness among women in southwestern Uganda: a community-based survey

**DOI:** 10.3402/gha.v7.22904

**Published:** 2014-01-09

**Authors:** Gustav Asp, Karen Odberg Pettersson, Jacob Sandberg, Jerome Kabakyenga, Anette Agardh

**Affiliations:** 1 Social Medicine and Global Health, Department of Clinical Science (Malmö), Lund University, Lund, Sweden; 2 Faculty of Medicine, Mbarara University of Science & Technology, Mbarara, Uganda

**Keywords:** birth preparedness, skilled birth attendant, mass media exposure, newspaper, radio, Uganda, low-income country

## Abstract

**Background:**

Exposure to mass media provides increased awareness and knowledge, as well as changes in attitudes, social norms and behaviors that may lead to positive public health outcomes. Birth preparedness (i.e. the preparations for childbirth made by pregnant women, their families, and communities) increases the use of skilled birth attendants (SBAs) and hence reduces maternal morbidity and mortality.

**Objective:**

The aim of this study was to explore the association between media exposure and birth preparedness in rural Uganda.

**Method:**

A total of 765 recently delivered women from 120 villages in the Mbarara District of southwest Uganda were selected for a community-based survey using two-stage cluster sampling. Univariate and multivariate logistic regression was performed with generalized linear mixed models using SPSS 21.

**Results:**

We found that 88.6% of the women surveyed listened to the radio and 33.9% read newspapers. Birth preparedness actions included were money saved (87.8%), identified SBA (64.3%), identified transport (60.1%), and purchased childbirth materials (20.7%). Women who had taken three or more actions were coded as well birth prepared (53.9%). Women who read newspapers were more likely to be birth prepared (adjusted OR 2.2, 95% CI 1.5–3.2). High media exposure, i.e. regular exposure to radio, newspaper, or television, showed no significant association with birth preparedness (adjusted OR 1.3, 95% CI 0.9–2.0).

**Conclusion:**

Our results indicate that increased reading of newspapers can enhance birth preparedness and skilled birth attendance. Apart from general literacy skills, this requires newspapers to be accessible in terms of language, dissemination, and cost.

Exposure to mass media has resulted in positive health outcomes regarding family planning, knowledge of HIV/AIDS, and a skilled birth attendant (SBA) at delivery (1–3). Birth preparedness, i.e. the preparations for childbirth undertaken by pregnant women, their families, and their communities, is a key recommendation of the World Health Organization (WHO) to reduce maternal mortality in low- and middle-income countries (4, 5).

Although the number of women dying during pregnancy, childbirth, and the immediate postpartum period has decreased globally from around 543,000 in 1990 to 287,000 in 2010 (6), the challenge of improving maternal survival is still enormous. Each pregnancy and every childbirth involves the risk of developing obstetric complications. Skilled care during pregnancy and childbirth, as well as access to emergency care, are therefore top priorities in order to reduce maternal mortality (7, 8). The proportion of deliveries assisted by SBAs is used as a proxy indicator for maternal deaths. The use of SBAs in low- and middle-income countries has increased from 55% in 1990 to 65% in 2010 (9). Generally, the increase has been slower in sub-Saharan Africa (SSA) but according to the Ugandan Demographic and Health Survey (UDHS) (10), Uganda has improved rapidly from 42% of deliveries being assisted by an SBA in 2006 to 58% in 2011.

The term birth preparedness is applied to signify important steps a mother may take to ensure a safe childbirth through skilled care. It includes factors such as saving money, identifying an SBA, finding a blood donor, and arranging transportation for delivery and any obstetric emergencies (11). The use of family/replacement blood donors is, however, discouraged by WHO due to the higher prevalence of transmission–transmitted infections (12, 13). Birth preparedness is considered to reduce delay in the decision to seek care (Delay I) and delay in reaching the health facility (Delay II), as presented in the three delays model that explores the chain of factors leading to maternal morbidity and mortality (14). Studies from Uganda, Burkina Faso, and India confirm that birth preparedness increases the likelihood of delivering with an SBA (15–17). It is an integral part of the spectrum of care for maternal and newborn health, which shifts from the implementation of specific interventions to holistic packages (4).

The health promoting effect of mass media can be transmitted through increased awareness and knowledge and changed attitudes, social norms, and behaviors (1). In the area of maternal health, for instance, high exposure to television and newspapers in northern Ghana was found to be associated with giving birth with the assistance of an SBA (3). In India, high exposure to mass media among women increased their use of antenatal care (ANC) services and the probability of being assisted by an SBA as well as giving birth at a health facility (18–20). Goli et al. argue that low exposure to mass media is one of the main pathways through which inequalities in maternal and child health is perpetuated in urban India (20).

Exposure to mass media varies largely within low-income countries, particularly when comparing the exposure to television and newspapers between urban and rural populations, the rich and the poor, and people with different education levels. In Uganda, 60% of the urban female population watch television at least once a week, but only 10% in rural areas. Newspapers are read at least once a week by 37% of the women with secondary level education and 9% of the women with primary education. Finally, 27% of the women in the two highest wealth quintiles read newspapers, as compared to 4% in the two lowest wealth quintiles, according to the 2012 UDHS (10). Radio, on the other hand, has a much broader coverage due to its greater ease of dissemination and the low cost on both the listener and the broadcaster sides (21). This is exemplified in Uganda where 73% of the rural women and 49% of those in the lowest wealth quintile listen to the radio at least once a week. In 2012, UNESCO reported that 75% of the households in low- and middle-income countries have access to radio (22).

In 2009, the Government of Uganda adopted a ‘Road Map for Accelerating the Reduction of Maternal and Neonatal Mortality and Morbidity’. Birth preparedness was identified as a key component in creating a demand for maternal health services and ensuring a continuum of care between the household and the health facility. Media outlets and practitioners were acknowledged as important stakeholders in delivering information on maternal and newborn health and promoting safe motherhood programs. They should therefore be actively involved in the process through public/private partnerships (23).

Earlier research has pointed out the importance of mass media in public health (1–3) and the association between media exposure and birth preparedness is an area of interest that has been given priority at the highest political level (23). To the best of our knowledge, no previous research has been conducted in Uganda on the impact media has had on a woman's preparation for childbirth. Consequently, the overall aim of this study was to explore the association between birth preparedness and exposure to media among women in the Mbarara District in southwest Uganda.

## Method

### Setting

The study was conducted in the Mbarara District in southwest Uganda. The district is divided in Mbarara Municipality, which is the urban center, and Kashari and Rwampara Counties which are mainly rural. The total population is 436,400 (24). The area contains 46 health centers with various levels of health services provision (II–IV) that all provide antenatal and emergency obstetric care, although Mbarara Regional Referral Hospital is the only one providing comprehensive obstetric care. Mbarara Municipality also has four private hospitals. Eighty percent of the Ugandan population lives in rural areas where the economy is predominately agricultural. According to the World Bank, 38% live in extreme poverty, i.e. below 1.25 USD per day in purchasing power parity (25). According to the 2012 UDHS, only 5% of the rural population has electricity in their homes compared to 55% of the urban population. The literacy rate among women in Uganda is 64.2%, but higher in the southwest (75.5%) (10).

The study was given ethical clearance by the Uganda National Council of Science and Technology and Lund University. Permission to conduct the study was also given by local leaders at the district, county, and village levels. A written consent form was read to each participant and a signature or thumbprint was obtained before the interviews.

### Sample and data collection

Participants in the community survey were selected by using a two-stage cluster sampling technique. In the first stage, 120 of the 699 villages in the study area were randomly chosen. The average population of a village is approximately 500. In each village, a starting point was alternately identified at the center or on the periphery with the help of a Village Health Team member. Two research assistants moved in opposite directions through the village stopping at every second household until 10 women who were either pregnant or had delivered within the last 12 months were identified. A total of 1,199 women were interviewed. Out of these, 765 had delivered during the last 12 months and were included in the study. The participants were equally divided between Kashari (50.8%) and Rwampara Counties (49.2%).

The women were asked about their knowledge, perceptions, and experiences regarding pregnancy, childbirth, and the postpartum period and about their media consumption and exposure to media interventions. The information was obtained by means of a Women's Safe Motherhood Questionnaire developed by the Johns Hopkins Program for International Education in Gynecology and Obstetrics (JHPIEGO) (11) that had been adapted to the Ugandan context and pretested in a neighboring district. After the pretesting, a question was added about the purchase of childbirth materials since it is an important preparation for childbirth in Ugandan. A total of 12 research assistants were recruited, each having a bachelor's degree in social science and previous experience in survey data collection. They all were trained for 1 week on the administration of the data instrument.

The data collection took place from September to December 2010 in Kashari County and from April to May 2011 in Rwampara County under the supervision of the principal investigator. The questionnaires were continuously verified for completeness and consistency by the field supervisors.

### Definition of variables

#### Socio-demographic and reproductive variables

*Area of residence* was coded into ‘Kashari’ or ‘Rwampara’ and ‘rural’ for villages or ‘semi-urban’ for trading centers. No urban areas existed in the two counties.

*Age* was coded into ‘<20 years’, ‘20–24 years’ and ‘≥25 years’.

*Marital status* was coded as ‘married’ for women who were married or in union and ‘not married’ for single, widowed, divorced or separated.

*Religion* was coded as ‘Christians’ for women belonging to the Roman Catholic, Church of Uganda or Seventh-day Adventists. The remaining women were coded as ‘Other’.

*Highest education level completed* was coded into ‘< primary school’, ‘primary school’ or ‘≥ secondary school’.

*Reading ability* was categorized based on the question ‘Can you read a letter, Bible, or a newspaper easily, with difficulty, or not at all?’ Easily was coded as ‘yes’ and with difficulty/not at all was coded as ‘no’.

*Travel time to health facility* was coded as ‘<1 hour’ and ‘≥1 hour’.

*Parity* was calculated by adding the reported number of live births and stillbirths and coded as ‘primipara’, ‘2–4’ or ‘≥5’ births.

*ANC visits* was coded as ‘<4’ or ‘≥4’ visits.

#### Media exposure variables

*Read a newspaper/listen to radio/watch television* was coded ‘yes’ or ‘no’ based on the question ‘Do you ever read newspaper/listen to radio/watch television?’

*Frequency of exposure* was coded as; ‘almost every day’, ‘at least once a week’ or ‘less than once a week’.

*Media exposure* was defined as ‘high’ if the women were exposed to radio or television ‘almost every day’ or read a newspaper ‘at least once a week’. The regional newspaper in the local language is a weekly paper, and so weekly newspaper exposure was considered ‘high media exposure’.

*Read/heard/saw information on birth preparedness in the past 6 months* was coded ‘yes’, ‘no’, or ‘don't know’.

*Exposure to birth preparedness information through newspaper/radio/television* was inquired into within those groups exposed to the separate media.

#### Birth preparedness variables

Four variables were used as measures for birth preparedness, as follows:

*Bought childbirth materials* was determined by asking ‘which arrangements did you or your family make for the birth of this child?’ and women who had bought a complete ‘Mama kit’ with the necessary materials for childbirth or any of these materials separately were coded as having bought childbirth materials.

*Saved money* was determined by asking ‘Did you or your family save money for the birth of this child?’.

*Identified transport to place of delivery* was determined by asking the question ‘Did you or your family identify transport for the birth of this child?’.

*Identified SBA for delivery* was determined by asking ‘Did you or your family identify a skilled provider for the birth of this child?’. Those women who delivered in a health facility and who, in a subsequent question, stated that this place of delivery was planned during the pregnancy, were also coded as having identified a SBA for delivery.

*Well birth prepared* was defined as having taken at least three of the four actions above.

### Statistical analysis

Sample size was predetermined by the existing database. Statistical analyses were conducted with SPSS Version 21 and all analyses accounted for the intra-cluster correlation. Generalized linear mixed models were used to calculate the odds ratios (OR) and 95% confidence intervals (CI) for the associations between media exposure and being well birth prepared (i.e. bought childbirth materials, saved money, identified transport to place of delivery, and identified SBA for delivery). Multivariate analyses included adjustments for age, education, location of residence, parity, travel time to health facility, and ANC visits.

## Results

The socio-demographic and reproductive variables are presented in Table 1. A majority of the women (83.9%) lived in a rural area, and 44.7% had a travel time of 1 hour or more to a health facility. One-third (34%) could not read at all or only with difficulty and 29.3% had not completed primary school. A total of 23% were primipara and 30.2% had a parity of five or more. Two-thirds (68.9%) had attended the recommended minimum of four ANC visits.

**Table 1:** Socio-demographic and reproductive characteristics of recently delivered Ugandan women (n=765)

Characteristics	Number (*n*)	%
County		
Kashari	389	50.8
Rwampara	376	49.2
Location of residence		
Rural	642	83.9
Semi-urban	123	16.1
Age (years)		
<20	56	7.3
20–24	247	32.3
25–29	226	29.5
30–34	125	16.3
≥35	111	14.5
Religion		
Christians	735	96.2
Other	29	3.8
Missing	(1)	
Marital status		
Married	727	95.0
Not married	38	5.0
Educational level		
<Primary school	224	29.3
Primary school	365	47.8
≥Secondary school	175	22.9
Missing	(1)	
Can read letter/Bible/newspaper		
Not at all	122	15.9
With difficulty	138	18.0
Easily	505	66.0
Parity		
1	176	23.0
2–4	358	46.8
≥5	231	30.2
ANC attendance		
<4 visits	234	31.1
≥4 visits	518	68.9
Missing	(13)	
Travel time to health facility		
<1 hour	419	55.3
≥1 hour	339	44.7
Missing	(7)	

Exposure to mass media is shown in Table 2. Although 33.9% of the women indicated they read newspapers, only 6.5% read a newspaper ‘almost every day’ and 41.2% read a newspaper ‘at least once a week’. By far the most popular form of media was radio, to which 88.6% of the women were exposed. In this group 90.3% listened to the radio almost every day, while only 4.9% reported ever watching television. In total 81.8% were exposed to radio or television almost every day or read a newspaper at least once a week and were therefore coded as having a high media exposure. The vast majority of this group consisted of radio listeners. Almost half (46.3%) said they had heard or seen some information on birth preparedness in the past 6 months. Among the radio listeners, 43.9% were exposed to birth preparedness information on the radio; among women reading newspapers the corresponding number was 11.4%.

**Table 2:** Proportion of recently delivered women reporting media exposure and exposure to birth preparedness information (n=765)

	Number (*n*)	%
Ever read a newspaper		
No	492	66.1
Yes	252	33.9
Missing	(21)	
Exposure to newspaper		
Almost every day	16	6.5
At least once a week	101	41.2
Less than once a week	128	52.2
Missing	(7)	
Ever listen to radio		
No	87	11.4
Yes	678	88.6
Exposure to radio		
Almost every day	602	90.3
At least once a week	52	7.8
Less than once a week	13	1.9
Missing	(11)	
Ever watch television		
No	721	95.1
Yes	37	4.9
Missing	(7)	
Exposure to television		
Almost every day	26	72.2
At least once a week	8	22.2
Less than once a week	2	5.6
Missing	(1)	
Read/heard/saw birth preparedness information in the past 6 months		
Yes	354	46.3
No	397	51.9
Don't know	14	1.8
Read birth preparedness information from written source	28	11.4
Missing	(7)	
Heard birth preparedness information on radio	294	43.9
Missing	(9)	
Saw birth preparedness information on television	10	27.0
Media exposure*		
Low	137	18.2
High	617	81.8
Missing	(11)	

* *Defined as exposure to radio or television almost every day or newspaper at least once a week.

Table 3 shows the distribution of the variables included in birth preparedness. One-fifth of the women (20.7%) had bought childbirth materials, 87.8% had saved money, 60.1% had identified transport for delivery, and 64.3% had identified a skilled provider or health facility for delivery. This resulted in a total of 53.9% of the women being well birth prepared.

**Table 3:** Reports of birth preparedness by recently delivered Ugandan women (n=765)

	Number (*n*)	%
Bought childbirth materials	158	20.7
Saved money	672	87.8
Identified transport	460	60.1
Identified skilled provider or health facility	492	64.3
Well birth prepared*	412	53.9

* Defined as having taken at least three of the four actions above.

Table 4 shows the results of univariate logistic regression analysis for the associations between socio-demographic and reproductive factors on the one hand, and birth preparedness on the other. The women who had completed secondary school were more likely to be well birth prepared (OR 1.9, 95% CI 1.2–3.0). Birth preparedness decreased with increasing parity: women who had given birth to five or more children were significantly less birth prepared than primiparas (OR 0.6, 95% CI 0.4–0.99). The women who attended at least four ANC visits were more birth prepared (OR 1.5, 95% CI 1.1–2.1).

**Table 4:** Associations (OR, 95% CI) between socio-demographic and reproductive variables, and being well birth prepared in a sample of recently delivered Ugandan women (n=765)

	Well birth prepared *n* (%)	OR (95% CI)
County		
Kashari	214/389 (55.0)	1.0 (ref)
Rwampara	198/376 (52.7)	0.9 (0.6–1.4)
Location of residence		
Rural	349/642 (54.4)	1.0 (ref)
Semi-urban	63/123 (51.2)	0.9 (0.6–1.5)
Age (years)		
<20	34/56 (60.7)	1.0 (ref)
20–24	136/247 (55.1)	0.7 (0.4–1.4)
≥25	242/462 (52.4)	0.6 (0.3–1.1)
Marital status		
Married	396/727 (54.5)	1.0 (ref)
Not married	16/38 (42.1)	0.6 (0.3–1.3)
Educational level		
<Primary school	102/224 (45.5)	1.0 (ref)
Primary school	199/365 (54.5)	1.4 (0.9–2.0)
≥Secondary school	110/175 (62.9)	1.9 (1.2–3.0)
Missing	(1)	
Can read letter/Bible/newspaper		
No	114/260 (43.8)	1.0 (ref)
Yes	298/505 (59.0)	1.8 (1.3–2.5)
Parity		
1	105/176 (59.7)	1.0 (ref)
2–4	193/358 (53.9)	0.8 (0.5–1.2)
≥5	114/231 (49.4)	0.6 (0.4–0.99)
ANC attendance		
<4 visits	110/234 (47.0)	1.0 (ref)
≥4 visits	292/518 (56.4)	1.5 (1.1–2.1)
Missing	(13)	
Travel time to health facility		
<1 hour	234/419 (55.8)	1.0 (ref)
≥1 hour	176/339 (51.9)	0.8 (0.6–1.1)
Missing	(7)	

Table 5 provides an analysis of the associations between media exposure and birth preparedness with unadjusted an adjusted ORs. An association was found between reading newspapers and being well birth prepared (OR 2.2, 95% CI 1.5–3.2). Exposure to a newspaper at least once a week did not strengthen the association (OR 1.7, 95% CI 1.03–2.8). Listening to the radio did not have a significant effect on birth preparedness (OR 1.3, 95% CI 0.8–2.2), nor did watching television (OR 0.7, 95% CI 0.3–1.5). Women with high media exposure were not birth prepared to a higher extent (OR 1.3, 95% CI 0.9–2.0).

**Table 5:** Associations (OR, 95% CI) between media exposure and being well birth prepared in a sample of recently delivered Ugandan women (n=765)

	Well birth prepared, *n* (%)	Unadjusted OR (95% CI)	Adjusted OR (95% CI)*
Ever read a newspaper			
No	233/492 (47.4)	1.0 (ref)	1.0 (ref)
Yes	169/252 (67.1)	2.2 (1.6–3.2)	2.2 (1.5–3.2)
Missing	(21)		
Exposure to newspaper			
Less than once a week or not at all	319/620 (51.5)	1.0 (ref)	1.0 (ref)
At least once a week	80/117 (68.4)	2.0 (1.2–3.1)	1.7 (1.03–2.8)
Missing	(28)		
Ever listen to radio			
No	39/87 (44.8)	1.0 (ref)	1.0 (ref)
Yes	373/678 (55.0)	1.5 (0.94–2.5)	1.3 (0.8–2.2)
Exposure to radio			
At least once a week, less often, or not at all	69/152 (45.4)	1.0 (ref)	1.0 (ref)
Almost every day	338/602 (56.1)	1.5 (1.01–2.2)	1.3 (0.8–1.9)
Missing	(11)		
Ever watch television			
No	391/721 (54.2)	1.0 (ref)	1.0 (ref)
Yes	19/37 (51.4)	0.8 (0.4–1.7)	0.7 (0.3–1.5)
Missing	(7)		
Exposure to television			
At least once a week, less often, or not at all	394/731 (53.9)	1.0 (ref)	1.0 (ref)
Almost every day	16/26 (61.5)	1.3 (0.5–3.1)	1.1 (0.4–2.8)
Missing	(8)		
Media exposure†			
Low	60/137 (43.8)	1.0 (ref)	1.0 (ref)
High	348/617 (56.4)	1.6 (1.1–2.4)	1.3 (0.9–2.0)
Missing	(11)		

* Adjusted for age, education, location of residence, parity, travel time to health facility, and ANC visits.

† Defined as exposure to radio or television almost every day or newspaper at least once a week.

## Discussion

Our findings show a significant relationship between reading newspapers and being birth prepared among rural women in southwest Uganda, regardless of the frequency of exposure. The women listening to the radio or watching television were not significantly more birth prepared. When comparing our results with the exposure to mass media for rural women reported in the 2012 UDHS, we found a higher proportion of women in our study were being exposed at least once a week to radio (85.5% vs. 73.2%) and newspapers (15.7% vs. 10.0%). Exposure to television on a weekly basis, on the other hand, was lower (4.5% vs. 9.8%) (10). Additionally, our data provided more detailed information on the frequency of exposure than the UDHS and could thus be used for the high vs. low media exposure variable. Since the newspaper in the local language is a weekly, there is a strong contextual indication to define reading a newspaper at least once a week as high media exposure. For television and radio, we required an almost daily exposure to be included among those highly exposed. However, because of the high proportion of women who listened to the radio almost every day, this definition resulted in a large group of highly media-exposed women that mainly consisted of radio listeners. We retained this definition since we wanted to study those who were highly media exposed as a collective group. With this definition we are acknowledging that with the current accessibility of radio in low-income countries, a great majority of the world's population is exposed to traditional media.

The most common preparation for childbirth in our study was saving money in anticipation of the birth. This result is similar to findings from Burkina Faso, Ethiopia, and India (83.3, 68.9 and 76.9%, respectively) (16, 17, 26). Approximately two-thirds of the women surveyed had identified a skilled provider or health facility for delivery, which is similar to findings from India (69.6%) but higher than Burkina Faso (43.9%) or Ethiopia (34.8%). Women in Uganda are instructed to bring a ‘mama kit’ or childbirth material to the health facility, but only one-fifth of the women in our study had bought childbirth material. This raises questions in regards to its availability and accessibility. Further assessment of the situation is required in order to verify this finding. The proportion of women who arranged for transportation shows large differences between SSA (Burkina Faso 46.1%, Nigeria 62.3%) and Asia (India 29.5%, Nepal 28%) (16, 17, 27, 28). Hence, birth preparedness and the practice of its different components vary considerably between continents, countries, and regions. As with all behavior change programs, it is crucial to know one's audience and their challenges so that one may adopt interventions relevant for a specific setting (29).

Despite an extensive literature search, no studies were identified that had researched possible associations between media exposure and birth preparedness. Our findings should therefore be considered in relation to other factors associated with media exposure. The exposure rate for different media varies greatly between continents and countries so that findings from one study cannot be easily translated to another setting. Most studies from Asia do not include newspapers in their media exposure variable. The findings of the Bangladesh Demographic and Health Survey show that the weekly exposure to radio (4.7%) and newspapers (6.3%) among women are substantially lower than in our study population, but the weekly exposure to television is 10 times higher (48.4%) (30). Not surprisingly, a study from Bangladesh exploring the association between media exposure and knowledge of HIV/AIDS reported that television was the most influential in source of transmission and prevention knowledge of AIDS. Exposure to newspapers also had a significant association with people hearing about AIDS, and was more associated with myth rejection than television. Radio, however, had a low impact on knowledge of HIV/AIDS in Bangladesh (2).

Findings from Ghana, with similar exposure to radio and newspapers as Uganda but a 10 times higher exposure to television (29), indicated that deliveries assisted by an SBA increased with the frequency of exposure to television. The same association appeared with regard to reading newspapers, although the significance did not remain after adjusting for confounders. However, as the variables adjusted for are not presented in the Ghanaian study, it is difficult to draw any conclusions regarding the association. The same study showed that exposure to radio had no association with being delivered by an SBA (3).

Our study and those from multiple other settings (17, 26) have demonstrated the positive effect of education and literacy in birth preparedness, identifying education as a major social determinant of health (31). However, no earlier studies on birth preparedness have shown the added value of reading newspapers. According to our findings, birth preparedness in Uganda is not discussed to any greater extent in the newspapers than on the radio, and therefore cannot explain the differences between the two groups. As stated in the African Media Barometer Uganda 2012, newspapers are expensive when viewed in relation to the income of many Ugandans (32). The level of household income might be an important confounder to this association. People who read newspapers might also be more likely to discuss issues with others, and such interpersonal interactions are considered important when initiating behavioral change (33). Further research is needed to explore how reading a newspaper may be associated with birth preparedness. A content analysis of Ugandan newspapers, preferably both quantitative and qualitative, would be needed for an in-depth understanding of the influence of newspapers (34). It would also facilitate further improvements in the maternal health information provided by mass media, as requested by the Ugandan government (23).

Although birth preparedness information was more frequent on the radio, listening to the radio did not make women significantly more birth prepared. On the other hand, as stated in the concept of health literacy, mere access to information is not enough: it needs to be understood and assimilated in order to lead to behavior change (35). This is especially true when the information conflicts with traditional practices and social norms, such as home deliveries, to which Ugandan women are accustomed (36). Radio generally has wider coverage and reaches vulnerable populations to a greater extent than other forms of media and may therefore be better used for the successful dissemination of interventions in the form of edutainment programs (37). However, apart from the many registered FM stations in Uganda (276 in 2011) (32), the country faces another challenge for attaining broad coverage with such interventions due to numerous local languages.

The increasing numbers of deliveries that take place in Uganda with the assistance of an SBA indicate an important change, although the maternal mortality ratio remains high (10). The ‘Road Map for Accelerating the Reduction of Maternal and Neonatal Mortality and Morbidity in Uganda’ (23) is a powerful document that has the potential to improve maternal health if appropriately implemented and adequately funded by the Ugandan government.

### Strengths and limitations of this study

By using two-stage cluster sampling and interviewing a relatively large number of recently delivered women for this study, a good estimate of birth preparedness and mass media exposure among women in rural southwest Uganda was obtained. Ideally a birth preparedness questionnaire should be given to women in late pregnancy in order to capture all preparations made prior to childbirth and avoid recall problems (38). However, this would lead to difficulties in obtaining the necessary sample size. A limit of 1 year was set in this study to minimize recall problems.

In this paper, we have only looked at the effect of direct exposure to mass media on our study group. However, communication researchers recognize the effect of exposure on the community as a whole. For some behaviors, a change can occur when a certain proportion of the community, rather than separate individuals, is exposed (1). When considering the effect of indirect exposure to mass media, a husband's exposure is likely to have the single most important influence on a woman. The importance of the husband in maternal and child health has been increasingly acknowledged and research has shown that a husband who takes an active role in this regard will increase his wife's birth preparedness (15, 39). We did not look at the husband's exposure to mass media, although it might have had an independent effect on birth preparedness.

## Conclusion

Our results indicate that birth preparedness, and ultimately skilled birth attendance, can be enhanced through increased reading of newspapers. Apart from requiring general literacy skills this mean that newspapers have to be accessible with regard to language, dissemination and cost.
